# Malaria increased the risk of stunting and wasting among young children in Ethiopia: Results of a cohort study

**DOI:** 10.1371/journal.pone.0190983

**Published:** 2018-01-11

**Authors:** Taye Gari, Eskindir Loha, Wakgari Deressa, Tarekegn Solomon, Bernt Lindtjørn

**Affiliations:** 1 School of Public and Environmental Health, College of Medicine and Health Sciences, Hawassa University, Hawassa, Ethiopia; 2 Centre for International Health, University of Bergen, Bergen, Norway; 3 Department of Preventive Medicine, School of Public Health, College of Health Sciences, Addis Ababa University, Addis Ababa, Ethiopia; Food and Drug Administration, UNITED STATES

## Abstract

**Introduction:**

Given the high prevalence of malnutrition in a malaria-endemic setting, improving nutritional status could serve as a tool to prevent malaria. However, the relationship between the two conditions remains unclear. Therefore, this study assessed the association between under-nutrition and malaria among a cohort of children aged 6 to 59 months old.

**Methods:**

Two cohorts of children were followed for 89 weeks in a rural Rift Valley area of Ethiopia. In the first approach (malaria-malnutrition), a cohort of 2,330 non-stunted and 4,204 non-wasted children were included to assess under-nutrition (outcome) based on their previous malaria status (exposure). In the second approach (malnutrition–malaria), a cohort of 4,468 children were followed-up to measure malaria (outcome), taking under-nutrition as an exposure. A weekly home visit was carried out to identify malaria cases. Four anthropometry surveys were conducted, and generalized estimating equation (GEE) method was used to measure the association between undernutrition and malaria.

**Results:**

The prevalence of stunting was 44.9% in December 2014, 51.5% in August 2015, 50.7% in December 2015 and 48.1% in August 2016. We observed 103 cases with 118 episodes of malaria, 684 new stunting and 239 new wasting cases. The incidence rate per 10,000 weeks of observation was 3.8 for malaria, 50.4 for stunting and 8.2 for wasting. Children with malaria infection, [Adjusted Odds Ratio (AOR) = 1.9; 95% Confidence Interval (CI), 1.2–2.9)] and younger age (AOR = 1.3; 95% CI, 1.1–1.5) were more likely to be stunted. Furthermore, children with malaria infection (AOR = 8.5; 95% CI, 5.0–14.5) and young age group (AOR = 1.6; 95% CI, 1.2–2.1) were more likely to be wasted. However, stunting and wasting were not risk factors of subsequent malaria illness.

**Conclusions:**

Malaria infection was a risk factor for stunting and wasting, but stunting or wasting was not associated with subsequent malaria illness. As our study shows that malaria is a risk factor for stunting and wasting, a close follow-up of the nutritional status of such children may be needed.

**Trial registration:**

PACT R2014 11000 882128 (8 September 2014).

## Introduction

Malaria is a public health problem in the developing world; particularly in sub-Saharan African countries where malaria kills a child every two minutes [1]. The disease remains one of the major challenge for people's health and livelihood around the world [1]. On the other hand, malnutrition is an underlying cause of death for approximately 45% of children under the age of five years [2]. Globally, 161 million children under five years old were stunted, and 51 million were wasted in 2012 [3, 4]. One-third of the world's stunted children were living in sub-Saharan Africa [5]. Malnutrition could refer both to under- and over-nutrition [6], but in this study we use the term to refer to under-nutrition (stunting, underweight and wasting).

In Ethiopia, malaria is a common cause of childhood illness [1, 7].Nearly, 68% of the land mass of the country have ecological characteristics favourable for malaria transmission, and about 60% of the population is at risk of malaria infection [8]. The transmission of malaria is seasonal and unstable [8]. *Plasmodium falciparum* (60%) and *P*.*vivax* (40%) are the two main causes of malaria [9]. According to the recent malaria indicator surveys, the national prevalence of malaria among children was 1.4% in 2011[7] and 0.6% in 2015 [10]. A pilot study conducted for the preparation of a community based malaria prevention trial in Adami Tulu district, Ethiopia (same study area with current study) in 2013, has shown a malaria incidence of 6.8 cases per 10,000 person weeks of observation among children [11]. In addition, under-nutrition is a major public health problem in the country. According to the 2016 Demographic and Health Survey (DHS), 47% of rural children were stunted and 10% were wasted [12–14].

Good nutrition and healthy growth during a period from conception to a child's second birthday—the 1,000 days—have lasting benefits throughout life, and undernourished children reaching this age could suffer from irreversible health problems [15]. Under-nutrition could be the result of poor dietary intake often combined with infectious disease [16]. On the other hand, malnutrition is a well-known underlying cause for many infectious disease-related causes of child deaths [17, 18]. Malaria and malnutrition co-exist in a setting where the two conditions are highly prevalent [19]. However, the relationship between malaria and under-nutrition is complex and remains unclear. Previous research shows mixed findings, e.g.: a community-based survey from Ghana showed underweight as a contributing risk to malaria infection [18], whereas results from a follow-up study and repeated cross-sectional surveys showed stunting as a contributing risk to malaria [20]. A case-control study from Ethiopia reported wasting as a contributing risk to malaria [21]. Some cohort studies showed a lower contributing risk to malaria infection among malnourished children [22–24], although results from other cross-sectional surveys did not report any association between malaria and malnutrition [25, 26]. Meanwhile, others observed malaria infection as a risk factor for under-nutrition [27–29]. In summary, most of the studies assessing the possible association between malaria and malnutrition were either institutional-based surveys that could not be generalizable [30], or community-based cross-sectional studies, which could have less strength of evidence to support a causal relationship [25]. Moreover, both case control [21] and cohort [31] studies show inconsistent findings.

Long-lasting insecticidal nets (LLINs), indoor residual spraying (IRS) and prompt diagnosis and treatment are the primary tools in reducing malaria-related illness and deaths [1]. In the meantime, evidence on the relationship between under-nutrition and malaria could also be used as an additional means to help the current malaria control activities. Given the high prevalence of malnutrition in a malaria-endemic setting, and if malnutrition is associated with malaria, improving nutritional status could serve as a tool to prevent malaria. However, in Ethiopia, there is a scarcity of follow-up studies measuring the relationship between the two conditions. Therefore, the general objective of this study was to assess the association between malaria and under-nutrition among a cohort of children aged 6 to 59 months old. The specific objectives were: 1) to assess the association between under-nutrition as an exposure and subsequent malaria infection, and 2) to evaluate the association between malaria as an exposure, and under-nutrition as outcome among children aged 6 to 59 months old.

## Materials and methods

### Study area

**Fig 1:**
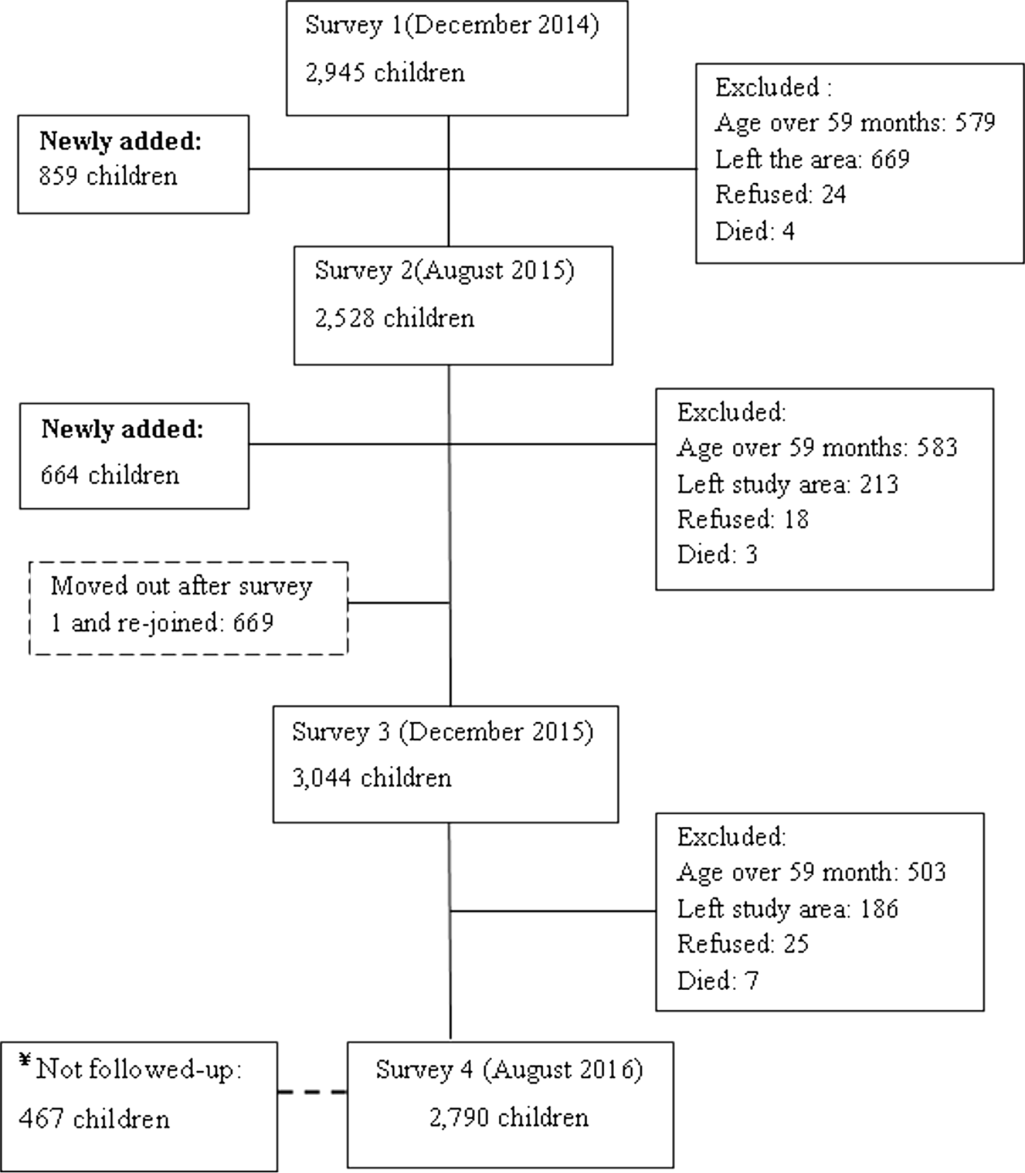
Study profile of children, in Adami Tullu District in south-central Ethiopia 2014–2016. ¥ The broken line to the left of the fourth survey box indicates newly joined children during the last survey (August 2016) and not followed for malnutrition. Thus, they were not included in the cohort study. However, they were included in the calculation of prevalence of undernutrition.

**Fig 2:**
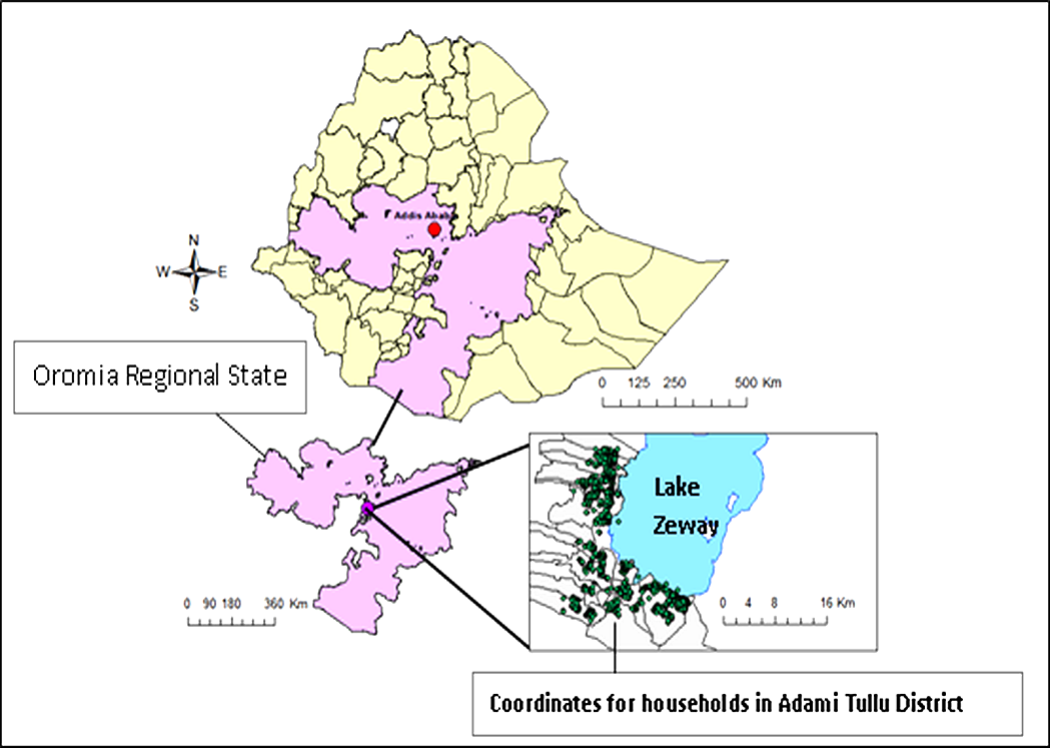
Map of the study area with location of households in Adami Tullu District in south-central Ethiopia. Re-print under a CCBY license, with permission from Deressa et al. Trials (2016).

The study profile of the participants was presented in Fig 1. The study was conducted in 13 *kebeles* (the lowest government administrative unit) in the Adami Tullu district, located 160 km south of Addis Ababa (Fig 2). According to the 2007 census, children under the age of five accounted for 12% of the total estimated population (147,000) of the district [32]. The total annual rainfall in 2014 (recorded for 8 months) was 673 mm, decreasing to 471 mm in 2015 [33]. The primary livelihood of the study population is based on rain-fed agriculture and livestock rearing, while cereal crops such as maize, wheat and teff are the main crops growing in the district. The study area was affected by repeated droughts and famines in the past few decades [34]. In 2015 and early 2016, the El Nino-triggered drought caused a serious food shortage [35], and the residents were getting food aid. Malaria and malnutrition were the major public health problems in the district [11, 36]. Each *Kebele* has one health post staffed by two community health extension workers. The health post provides basic health services, including the distribution of LLINs, the diagnosis of malaria with RDT and treatment with antimalarial drugs, as well as nutritional intervention, such as treatment for severe acute malnutrition and de-worming.

### Study design and participants

This study was a part of a malaria prevention trial, and the details have been presented in an earlier publication [37]. In brief, the trial was a cluster randomized controlled trial, and the unit of randomization was a village. The study villages were selected randomly from those within 5 km from Lake Zeway. The trial was based on four arms: LLINs + IRS, LLINs alone, IRS alone and control arms.

In this study on the association between malaria and under-nutrition, two cohort studies were followed-up from December 2014 to August 2016 with weekly visits. In the first cohort (called the malaria-malnutrition cohort), malaria was the main exposure and under-nutrition was the outcome. In this cohort, 2,330 non-stunted and 4,204 non-wasted children were considered as the study subjects (supplementary "S1 Fig and S2 Fig"). In the second cohort (called malnutrition–malaria cohort), a cohort of 4,468 children was enrolled using anthropometry surveys, and followed to determine malaria incidence (outcome) based on nutritional status (exposure).

### Sample size estimation

The sample size calculation for the main trial has been presented elsewhere[37]. In brief, the calculated sample size was 44 villages per four arms (LLINs+ IRS, LLINs alone, IRS alone and control arm), with each village having approximately 35 households. Overall, roughly 31,000 people in 6,072 households were included in the trial. In this study on the association between malaria and malnutrition, we followed all children (4468 children) aged 6 to 59 months who participated in the main trial. The total number of non-stunted children was 2,330, and the number of anthropometric measurements ranged between 1 and 4 for each child. Whereas, the total number of non-wasted children was 4,204, and the number of anthropometric measurements ranged from 1 to 4 for each child.

### Data collection

A household census was conducted to collect data on demographic and socioeconomic variables using a pre-tested interviewer-administered structured questionnaire. The data collectors were diploma graduate personnel. We carried out weekly home visits searching for children with a history of fever over the past 48 hours. The identified cases were referred to health posts for a malaria diagnosis. In addition, the families were advised to visit the health post between the visit days if their child developed a fever.

### Malaria diagnosis

According to the World Health Organization (WHO) recommendation, the two methods currently considered suitable for routine patient management are light microscopy and RDT [38]. The gold standard for over a century, light microscopy is useful to identify the *Plasmodium* parasite presence, species and accurate parasite counting (identification of high parasite density). However, high quality light microscopy requires well-trained, skilled staff, good staining reagents and electricity to power the microscope [38]. Whereas, rapid diagnostic tests (RDTs) based on lateral flow immune-chromatography, which can be done with little training, and have made malaria diagnostic tests accessible to the larger community [39]. In Ethiopia, light microscopy is used to diagnose malaria at the health centers and hospitals, whereas, at community health posts, the diagnosis of malaria is made using rapid diagnostic testing [40]. For the current study, we used RDT for malaria diagnosis. However, RDT does not measure malaria parasite counts. Therefore, we did not assess the association between malaria parasiteamia or severity and malnutrition.

At the health post, capillary blood samples were collected through a finger prick, and malaria was diagnosed using a multispecies *P*.*falciparum* and *P*.*vivax* RDT Care Start ^TM^ produced by Premier Medical Corporation Limited in India.

### Anthropometry survey

We conducted four anthropometric surveys in December 2014, August 2015, December 2015 and in August 2016. A standard wooden board was used to measure height or length, and a calibrated Salter spring scale was used to measure the weight of the children. The data collectors were trained, the instrument was standardized in the field work and the inter-rater reliability of the tools were analyzed [41]. The intra-technical error of measurement was 0.08 kg for weight and 0.14 cm for height, whereas the inter-technical error of measurement was 0.1kg for weight and 0.2 cm for height. The weight measuring scale was adjusted, and the weight was read to the nearest 0.1 kg. The length of children less than 24 months old was taken in the recumbent position, while for the older children the height was measured standing on a vertical measuring wooden board, which was read to the nearest 0.1 cm. Using the 2006 WHO multi-center growth reference study [42], weight for height (WHZ), height for age (HAZ) and weight for age (WAZ) were calculated. The children were classified as wasted with a WHZ < -2 Z score, stunted with a HAZ < -2 Z score and underweight with a WAZ < -2 Z score.

### Statistical analysis

Data were entered into IBM SPSS version 21 (SPSS Inc, Chicago, USA), and analyzed using STATA version 14 (StataCorp, Texas, USA). Descriptive statistics were used to summarize the data, and nutritional indices were calculated using the Emergency Nutrition Assessment (ENA) for SMART software 2011 [43]. A household wealth index was constructed using principal component analysis technique [44]. We included 14 variables that were related to household assets and livestock ownership [45], and the constructed index was used to categorize the households into three socio-economic classes, including rich, middle and poor.

The prevalence of under-nutrition was calculated for each survey, whereas the incidence of stunting, wasting and malaria was calculated per person-weeks of observation.

In the malaria-malnutrition cohort, the outcome variables stunting and wasting were assessed using anthropometric measurements. Because we only did anthropometry twice a year, it was difficult to capture the actual start of stunting or wasting, as the time of start of stunting or wasting could have occurred earlier than the time at diagnosis. Therefore, the use of time from malaria diagnosis to stunting or wasting diagnosis as an endpoint could result in underestimation of the incidence rate ratio of stunting or wasting, and could limit the use of Cox regression. On the other hand, the standard logistic regression could not allow for the repeated measures of the outcome variables, and could underestimate the standard error. However, the generalized estimating equations (GEE) procedure extends the generalized linear model to allow for analysis of repeated measurements [46]. The repeated observation within one subject are not independent of each other, and therefore, GEE helps to correct for these within-subject correlations. In this study, stunting or wasting (outcome) was measured four times for a child, and the variable stunting or wasting was dichotomous that follows a binomial distribution. Thus, a logistic generalized estimating equation (GEE) was used to allow the repeated measures for the outcome variables, stunting or wasting. The specified probability distribution was binomial with logit link function and the working correlation matrix structure was exchangeable. The covariance matrix was robust estimator, and the scale parameter was Person chi-square (χ^2^). A hybrid with a maximum Fisher scoring iteration of 1 was used as a parameter estimation method. The main effect was the term used to build the reported model, and Kernel was specified for the log quasi-likelihood function. Child age, gender, malaria infection, wealth index, education of the household head and intervention arms were considered as the potential risk factors of stunting and wasting for each child. Lastly, the bivariate and multivariate logistic regression analysis were carried out and odds ratio was reported.

In the undernutrition-malaria cohort, the outcome variable, malaria case was assumed to follow a binomial distribution. The main exposure variables wasting and stunting were measured twice yearly and the outcome malaria variable (malaria) was identified through weekly home visit and patient self-referral between the visit days. Hence to account for within subject measurement correlations, a logistic GEE model was fitted taking into account child age, gender, stunting, wasting, wealth index, education of the household head and intervention arm as the potential risk factors of malaria cases for each child. To construct the model the following were specified: the scale parameter was Pearson χ^2^, the scale weight variable was the number of weeks a child had been observed, the covariance matrix was robust estimator and a hybrid with maximum Fisher scoring iteration of 1 as parameter estimation method. The specified log-likelihood function was Kernel, and the main effect was the term used to build the model. Bivariate and multivariate analysis were done to measure the risk factors of malaria, and odds ratio was reported.

### Ethical issues

Ethical clearance was obtained from the Institutional Review Board of the School of Public Health at Addis Ababa University, the National Ethical Committee of the Ministry of Science and Technology in Ethiopia (ref: 3.10/446/06) and the Regional Committee for Medical and Health Research Ethics, Western Norway (ref: 2013/986/REK Vest). A written permission letter was obtained from the Oromia Regional Health Bureau, East Shewa Zonal Health Department and the Adami Tullu District Health Office. As the majority of the study population cannot read and write, we had a challenge to get written consent. Thus, a verbal consent was obtained from the parents or caretakers before collecting the blood samples and anthropometric measurements of the children. A standard information sheet was used to explain the purpose of the study, and the participants were informed that participation was voluntary and that they had the right to withdraw any time during the study. They were assured that refusal to participate in the study would not affect their health service utilization at the health posts. This information was read to them using the information sheet in their own language, and their consent was recorded using check (√) mark. We also strictly supervised the data collectors, whether they are following the information sheets or not. Later, this document was stored at our field research station. Those children who were positive for malaria, and severely acutely malnourished, were treated according to the national treatment guidelines [40, 47]. Accordingly, those with *P*. *falciparum* or a mixed infection were given Artemether-Lumefantrin, while children with *P*. *vivax* were treated with Chloroquine.

## Results

### Participants and prevalence of under-nutrition

The mean (SD) age of the children in months was 33.6 (14.4) in December 2014, 37.4 (15.0) in August 2015, 35.6 (14.8) in December 2015 and 36 (15.2) in August 2016 surveys. Nearly 49% of them were girls in all the surveys (Table 1).

**Table 1:** Characteristics and nutritional status of children in Adami Tullu District in south-central Ethiopia, 2014–2016.

Variables		December 2014 (n = 2,945)	August 2015 (n = 2,528)	December 2015 (n = 3,044)	August 2016 (n = 2,790)
		Number (%)	Number (%)	Number (%)	Number (%)
**Gender**					
Boy		1,497 (50.8)	1,302 (51.5)	1,538 (50.5)	1,419 (51.9)
Girl		1,448 (49.2)	1,226 (48.5)	1,506 (49.5)	1,371 (49.1)
**Age in months**					
6–35		1,461 (49.6)	1,105 (43.7)	1,462 (48.0)	1,274 (45.7)
36–59		1,484 (50.4)	1,423 (56.3)	1,582 (52.0)	1,516 (54.3)
**Household Head education**					
Illiterate		1,689 (57.4)	1,465 (58.0)	1,794 (58.9)	1,624 (58.2)
Primary		936 (31.8)	775 (30.7)	916 (30.1)	855 (30.6)
Secondary and above		320 (10.8)	288 (11.3)	334 (11.0)	311 (11.2)
**Wealth status**					
Poor		945 (32.0)	801 (31.7)	1,045 (34.3)	961 (34.4)
Middle		1,000 (34.0)	875 (34.6)	1,015 (33.3)	942 (33.8)
Rich		1,004 (34.0)	852 (33.7)	984 (32.4)	887 (31.8)
**Intervention arm**					
IRS + LLIN		741 (25.2)	707 (28.0)	797 (26.2)	739 (26.5)
LLIN alone		752 (25.5)	569 (22.5)	784 (25.8)	734 (26.3)
IRS alone		670 (22.8)	541 (21.4)	679 (22.3)	630 (22.6)
Routine		782 (26.5)	711 (28.1)	784 (25.7)	687 (24.6)
**Anthropometric indicators**					
Median (IQR)	HAZ	-1.8 (-2.8- -0.8)	-2.1 (-3.2- -0.9)	-2.0 (-2.9- -1.1)	-1.9 (-2.8- -1.0)
	WAZ	-1.0 (-1.7- -0.4)	-1.8 (-1.1- -0.3)	-1.05 (-1.7- -0.4)	-1.12 (-1.7- -0.4)
	WHZ	-0.07 (-0.1–0.02)	0.2 (0.1–0.3)	0.1 (0.06–0.2)	-0.01 (-0.8–0.7)

IQR: Interquartile Range; IRS: Indoor Residual Spraying; LLINs: Long Lasting Insecticidal Nets

HAZ: Height-for-Age; WAZ: Weight for Age; WHZ: Weight-for-Height

**Fig 3:**
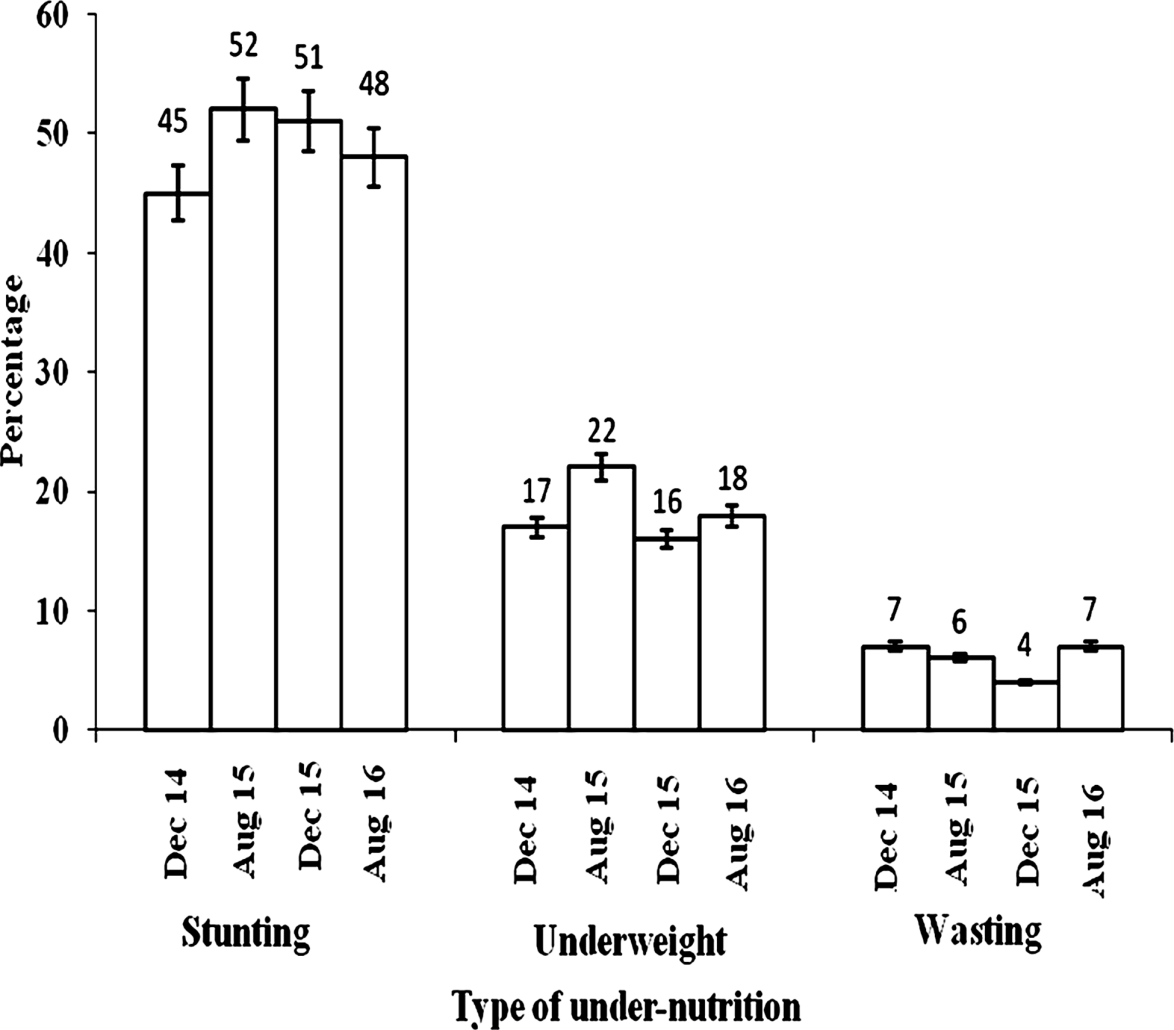
Prevalence of under-nutrition among children in Adami Tullu District in south-central Ethiopia, 2014–2016. I: Bar with 95% confidence level.

The overall prevalence of stunting was 44.9% in December 2014, 51.5% in August 2015, 50.7% in December 2015 and 48.1% in August 2016. The prevalence of underweight was 17.2% in December 2014, 21.7% in August 2015, 16.3% in December 2015 and 18.7% in August 2016, while the prevalence of wasting was 7.2% in December 2014, 5.7% in August 2015, 4.0% in December 2015 and 6.9% in August 2016 (Fig 3).

#### Prevalence of stunting and wasting: Correlation with prevalence of malaria

**Fig 4:**
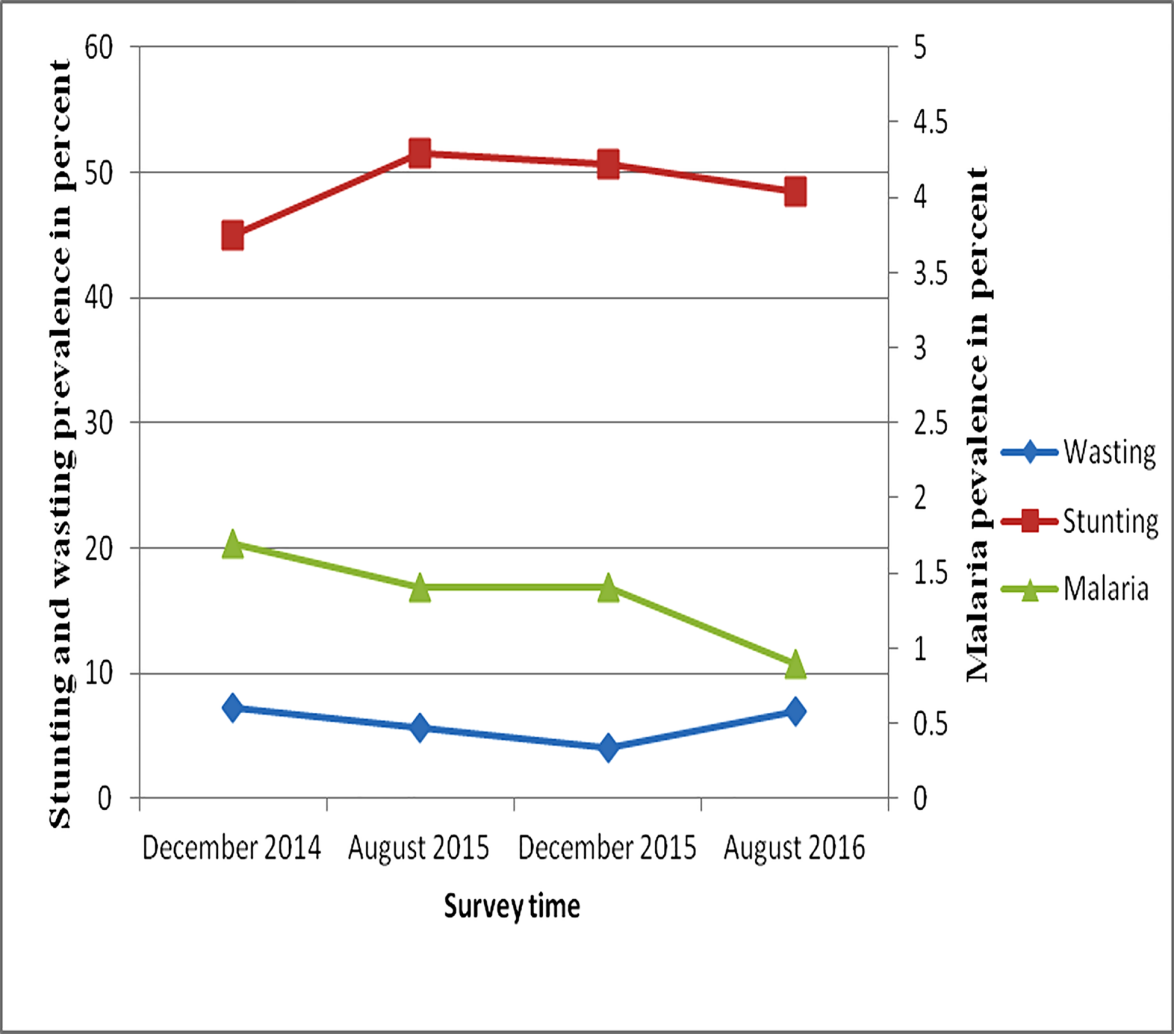
Prevalence of stunting, wasting and period prevalence of malaria among children in Adami Tullu District in south-central Ethiopia, 2014–2016.

As shown in Fig 4, stunting increased, but malaria prevalence decreased during the second and third surveys compared to the first survey (December 2014). We observed no significant correlation between prevalence of stunting and malaria (Spearman's correlation coefficient was -0.32, P-value = 0.684), and also no significant correlation between wasting and malaria (Spearman's correlation coefficient was 0.31, P-value = 0.68).

### Malaria-malnutrition cohort

#### Incidence of stunting

We observed 684 new stunting cases during the 89 weeks of follow-up; of which, 285 (41.7%) were severely stunted (HAZ <-3 z-score). Stunting was highest in the age group from 23–35 months (46%), followed by the age group 6–23 months (28.4%) and those above 36 months old (26.5%). The incidence rate of stunting was 50.3; (95% CI, 46.7–54.3) per 10,000 person-weeks of observation. The stratified analysis of stunting by malaria status and age group showed that age was not an effect-measure modifier of malaria effect (a risk ratio of 1.0 among children aged 6–23 months, 0.8 in the age group above 24 months, an overall crude risk ratio of 0.9 and an adjusted risk ratio of 0.9).

#### Risk factors of stunting

The fitted GEE model showed that younger age (AOR = 1.3; 95% CI, 1.1–1.5) and malaria infection (AOR = 1.9; 95% CI, 1.2–2.9) were risk factors of stunting after adjusting for the previous height-for-age (6 months preceding anthropometry survey). However, wealth status, education of household head, gender and intervention arms were not found to be the risk factors for stunting (Table 2).

**Table 2:** GEE model for stunting in children living in Adami Tullu District in south-central Ethiopia, 2014–2016.

Variables (N = 9320)		Unadjusted OR (95% CI)	Adjusted OR (95% CI)	P-value
Gender	Boy	1.0 (0.9–1.2)	1.1 (0.9–1.3)	0.23
	Girl	1	1	
Age in months	6–35	1.3 (1.1–1.4)	1.3 (1.1–1.5)	0.002*
	36–59	1	1	
Malaria infection¥	Positive	1.9 (1.2–2.9)	1.9 (1.2–2.9)	0.01*
	Negative	1	1	
Previous height-for-age†	<-2Z-score	12.0 (9.5–15.3)	12.3 (9.8–15.8)	<0.001*
	≥-2Z-score	1	1	
Wealth Status	Poor	1.2 (1.0–1.5)	1.2 (0.9–1.5)	0.13
	Middle	1.1 (0.9–1.3)	1.1 (0.9–1.3)	0.51
	Rich	1	1	
Education: household head	No formal	1.1 (0.8–1.5)	1.1 (0.9–1.5)	0.42
	Primary	1.4 (1.1–1.8)	1.3 (1.0–1.8)	0.05
	Secondary and above	1	1	
Intervention arm	IRS+ LLINs	1.1 (0.9–1.5)	1.1 (0.8–1.4)	0.4
	IRS alone	1.2 (0.9–1.5)	1.1 (0.9–1.5)	0.39
	LLINs alone	1.0 (0.8–1.3)	1.0 (0.7–1.2)	0.91
	Routine	1	1	

**¥:** malaria illness in the previous months preceding anthropometry survey

†: Height-for-age 6 months preceding anthropometry survey

OR: Odds Ratio; CI: Confidence Interval

*: P <0.05

IRS: Indoor Residual Spraying; LLINs: Long Lasting Insecticidal Nets

#### Incidence of wasting

Overall, 239 new wasting cases were registered, with severe wasting (WHZ<-3Z-score) accounting for 31% (74/239). The incidence rate of wasting was 7.2, (95% CI, 6.3–8.1)/10,000 person-weeks of observation.

#### Risk factors of wasting

In the GEE analysis, children in the younger age group (AOR = 1.6; 95% CI, 1.2–2.1) and children with malaria illness (AOR = 8.5; 95% CI, 5.0–14.5) were more likely to be wasted after adjusting for the previous weight-for-height (6 months preceding anthropometry survey). Nonetheless, education of household head, wealth status of the family, gender and intervention arms were not associated with wasting (Table 3).

**Table 3:** GEE model for wasting in children living in Adami Tullu District in south-central Ethiopia, 2014–2016.

Variable (N = 16,804)		Unadjusted	Adjusted	
		OR (95% CI)	OR (95% CI)	P-value
Gender	Boy	1.2 (0.9–1.5)	1.2 (0.9–1.5)	0.25
	Girl	1	1	
Age in months	6–35	1.5 (1.2–2.0)	1.6 (1.2–2.1)	0.001*
	36–59	1	1	
Malaria infection¥:	Positive	8.0 (4.7–13.3)	8.2 (5.0–14.3)	<0.001*
	Negative	1	1	
Previous weight-for-height†	<-2Z-score	2.4 (0.9–5.9)	2.3 (0.8–5.8)	0.45
	≥-2Z-score	1	1	
Wealth Status	Poor	0.9 (0.7–1.3)	0.9 (0.7–1.3)	0.66
	Middle	0.9 (0.7–1.3)	0.9 (0.6–1.2)	0.51
	Rich	1		
Education: household head	No formal	1.0 (0.7–1.6)	1.1 (0.7–1.6)	0.74
	Primary	0.9 (0.6–1.5)	1.0 (0.6–1.5)	0.83
	Secondary and above	1	1	
Intervention arm	IRS+LLINs	0.9 (0.6–1.3)	0.9 (0.6–1.3)	0.45
	IRS alone	0.9 (0.6–1.3)	0.9 (0.6–1.3)	0.63
	LIINs alone	1.0 (0.7–1.5)	1.0 (0.7–1.4)	0.89
	Routine	1	1	

**¥:** malaria illness in the previous months preceding anthropometry survey

†: Weight-for-height 6 months preceding anthropometry survey

OR: Odds Ratio; CI: Confidence Interval

*: P <0.05; IRS:

Indoor Residual Spraying; LLINs: Long Lasting Insecticidal Nets

#### Malnutrition-malaria cohort

Overall, 103 malaria cases with a total of 118 episodes of malaria were observed between December 2014 and August 2016. Of all children diagnosed with malaria, 12 children had more than one malaria episode. Slightly over half (53%) of the malaria cases were due to *P*. *falciparum*, followed by *P*. *vivax* (36%) and mixed infections (11%). The incidence of malaria was 3.8 (95% CI, 3.1–4.6)/10,000 person-weeks of observation.

#### Risk factors of malaria

The logistic GEE model showed that being in the poorest families AOR = 3.3; 95% CI, 1.7–6.3) were more at risk of malaria compared to children in rich families. However, stunting, wasting, gender, age, education of head of household and intervention arms were not associated with increased risk for malaria (Table 4).

**Table 4:** GEE model for malaria in children living in Adami Tullu District in south-central Ethiopia, 2014–2016.

Variable (N = 16,720)		Unadjusted OR (95% CI)	Adjusted OR (95% CI)	P-value
Gender	Boy	0.9 (0.5–1.4)	1.0 (0.6–1.6)	0.93
	Girl	1	1	
Age in months	6–35	1.1 (0.7–1.9)	1.1 (0.7–1.9)	0.62
	36–59	1	1	
Height-for-age	<-2Z-score	1.2 (0.8–2.0)	1.0(0.6–1.6)	0.89
	≥-2Z-score	1	1	
Weight-for-height	<-2Z-score	0.9 (0.4–2.0)	0.9 (0.4–1.4)	0.80
	≥-2Z-score	1	1	
Wealth index	Poor	3.5 (1.9–6.6)	3.3 (1.7–6.3)	0.002*
	Middle	1.9 (0.9–3.8)	1.8 (0.9–3.6)	0.12
	Rich	1	1	
Education: household head	No formal	0.7 (0.4–1.5)	0.8 (0.3–1.7)	0.52
	Primary	1.1 (0.5–2.2)	1.0 (0.4–2.4)	0.92
	Secondary and above	1	1	
Intervention arms	IRS+LLINs	1.5 (0.8–2.9)	1.6 (0.8–3.2)	0.18
	IRS alone	1.5 (0.8–2.9)	1.5 (0.7–3.2)	0.26
	LIINs alone	1.1 (0.5–2.3)	1.2 (0.5–2.6)	0.72
	Routine	1	1	

OR: Odds Ratio; IRS: Indoor Residual Spraying; LLINs: Long Lasting Insecticidal Nets

*: P <0.05

CI: Confidence Interval; IRR: Incidence Rate Ratio

## Discussion

In this study, malaria infection in the six months preceding the anthropometry survey was a risk factor for stunting and wasting. Even so, stunting or wasting did not contribute to an increased risk of malaria infection.

Our study was based on data from a large cohort of children recruited from a rural community, and the follow-up was for a relatively longer period of time (89 weeks). The continuous supply of Artemether-Lumefantrin and Chloroquine, the availability of a dedicated project staff in the health post and weekly active searches for cases motivated the residents to seek early diagnosis and treatment for malaria.

In our cohort studies the outcome variable is repeatedly measured; that means the outcome variable (stunting, wasting and malaria) is measured in the same child on several different occasions (four cross-sectional studies for undernutrition and weekly for malaria). In such studies the observations of one child over time are not independent of each other, and thus it is necessary to apply GEE method to fit logistic regression model, which consider the fact that the repeated measures of each child are corrected [46]. The outcome and main exposure variables (malaria and undernutrition) were measured using objective standard measurement tools.

The observed malaria incidence (3.8/10,000 person-weeks of observation) in this study was lower than that of a previous study from the same area in 2013 (6.8/10,000 child-weeks of observation) [11]. This may be related to reduced rainfall due to the severe drought that affected the study area during our study period [35]. During the drought season, the decrease in rainfall and increase in temperature could decrease the vector density and result in reduced malaria occurrence [48]. The observed stunting prevalence of 51.5% in August 2015, 50.7% in December 2015 and 48% in August 2016 in this study was higher than a study from Eastern Ethiopia (45.8%) [49] and South-West Ethiopia (40.4%) [25]. This could be due to the El Nino triggered severe drought and food shortage that affected large parts of Ethiopia [35].

In this study, malaria infection preceding an anthropometry survey was a risk factor for wasting. This could be explained by the potential effect of malaria to influence the host’s nutrition. Malaria could cause an acute weight loss through decreased food intake, and an increased energy requirement related to illness [50, 51]. Furthermore, children with a malaria infection need more protein and calories than uninfected children for rapid recovery, as inadequate food availability could increase the risk factors for under-nutrition. This finding is supported by a comparable cohort study from Vanuatu Island [28].

In addition, our data show that malaria infection preceding the malnutrition survey was a risk factor for stunting, and this finding was consistent with a cohort study from Kenya [27] and Ghana [29], which show a higher risk of stunting among children with malaria. However, a cohort study from Benin [52] did not observe an association between malaria and a subsequent occurrence of malnutrition. This could be due to a difference in study setting, in which the Benin study was an institutionally based that could be affected by selection bias.

It is well established that under-nutrition weakens the immune system, putting the child at more risk for infectious diseases, such as diarrhea, measles and respiratory infections [53, 54]. However, concerning the relationship between malnutrition and malaria, different studies showed inconsistent findings. Unlike our findings, studies from Kenya and Ghana observed a higher risk of malaria among children with malnutrition compared to those without malnutrition [18, 20]. This could be due to a difference in study design, in which these two studies used a cross-sectional design that could have less strength to establish the temporal relationship between the two conditions. A case-control study from Ethiopia reported a higher odds of malaria among severely wasted children [21], and this could be explained by a difference in the selection of the cases and controls. In such circumstances, the cases were not comparable with the controls, and could result in biased findings. Moreover, the use of institutional records (incomplete or inaccurate) to assess the exposure status could distort the true association in the path of causality. Nevertheless, another community-based cross-sectional survey in Ethiopia did not show malnutrition as a risk marker for malaria [25]. Additionally, a study from Ghana [29] demonstrated a higher impact of malnutrition on malaria-related deaths among children, though they did not observe malnutrition as a risk marker for malaria incidence, thereby supporting our findings. A systematic review of observational studies [55] observed that most of the studies did not show malnutrition as a risk marker of malaria incidence, but it could have a negative impact on malaria severity and death. In the same review, it was observed that malaria was a risk factor for malnutrition, which was in line with our study. On the contrary, studies by Ahmed et al.[23] and Mitangala et al.[24] observed a lower risk of malaria parasitemia among children with severe malnutrition. In our study, we used the RDT results and we did not assess the relationship between malnutrition and parasitemia or malaria by severity category.

This study had some limitations. The observed malaria incidence in this study (3.8/10,000 person-weeks) was lower than findings from the same area (8/10,000 person-weeks among the general population and 11/10,000 person-weeks among children) prior to our trial [11]. The lower malaria incidence could mainly be due to the decrease in rainfall and an increase in temperature during the trial period. In Ethiopia, a high prevalence of asymptomatic malaria infection was reported [56, 57], and studies also showed a high prevalence of anemia among asymptomatic plasmodium carriage [58]. In the current study, we did not assess asymptomatic malaria infection. In rural Ethiopia, only 57% of the infants were exclusively breast fed [59], nearly 50% of households were food insecure [60] and over half of children were infected with intestinal helminthes such as hookworm and schistosomiasis [61, 62]. Even so, we did not collect data on food security, breast feeding practices, micronutrient deficiencies and the occurrence of intestinal helminths infections such as hookworm and schistosomiasis that could contribute to increased prevalence of under-nutrition. Our previous work from same populations [45] showed a high anemia prevalence, and that stunting and malaria were contributing to the risk of anemia.

In conclusion, the data showed that malaria was a risk factor for stunting and wasting. Meanwhile, neither stunting nor wasting was associated with increased risk of malaria infection. As our study shows that malaria is a risk factor for subsequent stunting, a close follow-up of the nutritional status of such children may be needed.

## Supporting information

S1 Fig Study profile of non-stunted children in Adami Tullu District in south-central Ethiopia 2014–2016. ¥: Newly added include newborn children aged 6 month and above during the survey and newcomers.
(TIF)

S2 Fig Study profile of non-wasted children in Adami Tullu District in south-central Ethiopia, 2014–2016. ¥: Newly added include newborn children aged 6 month and above during the survey and newcomers.
(TIF)

## References

[1] WHO. World malaria report. Geneva, Switzerland: World Health Organization, 2015.

[2] WHO. Children: Reducing mortality: Geneva, Switzerland: World Health Organization; 2015

[3] WHO. Global nutrition targets 2025, Wasting policy brief. Geneva, Switzerland: World Health Organization; 2012.

[4] WHO. Global nutrition targets 2025, Stunting policy brief. Geneva, Switzerland: World Health Organization; 2012.

[5] WHO. The African Regional Health Report. World Health Organization Regional Office for Africa, 2014.

[6] WHO. Malnutrition: Quantifying the health impact at national and local levels, Geneva, Switzerland: World Health Organization; 2005.

[7] MOH. Ethiopian national malaria indicator survey. Ministry of Health, 2012.

[8] MOH. An epidemiological profile of malaria in Ethiopia Addis Ababa: Ethiopian Public Health Institute, Ministry of Health, 2014.

[9] USAID. President’s malaria initiatives Ethiopia. 2016.

[10] MOH. Ethiopia national malaria Indicator survey 2015. Addis Ababa: Ethiopian Public Health Institute, 2016.

[11] Gari T, Kenea O, Loha E, Deressa W, Hailu A, Balkew M, et al Malaria incidence and entomological findings in an area targeted for a cluster -randomized controlled trial to prevent malaria in Ethiopia: results from a pilot study. Malar J. 2016;15:145 doi: 10.1186/s12936-016-1199-4 26957044 10.1186/s12936-016-1199-4 PMC4784280

[12] Alemayehu M, Tinsae F, Haileslassie K, Seid O, G/egziabher G, Yebyo H. Nutritional status and associated factors among under-five children, Tigray, Northern Ethiopia. International Journal of Nutrition and Food Sciences 2014;3(6):579–86

[13] Medhin G, Hanlon C, Dewey M, Alem A, Tesfaye F, Worku B, et al Prevalence and predictors of undernutrition among infants aged six and twelve months in Butajira, Ethiopia: The P-MaMiE Birth Cohort. BMC Public Health. 2010;10:27 doi: 10.1186/1471-2458-10-27 20089144 10.1186/1471-2458-10-27 PMC2826285

[14] CSA. Ethiopian demographic and health survey. Central Statistical Agency: 2011.

[15] Black RE, Victora CG, Walker SP, Bhutta ZqA, Christian P, Onis Md, et al Maternal and child undernutrition and overweight in low-income and middle-income countries. The Lancet. 2013; 382 427–451. 10.1016/S0140-6736(13)60937-X 23746772

[16] Bryce J, Coitinho D, Darnton-Hill I, Pelletier D, Pinstrup-Andersen P. Maternal and child undernutrition: effective action at national level. The Lancet. 2008;371:510–26. 10.1016/S0140-6736(07)61694-8 18206224

[17] Caulfield Laura E, Onis Md, Blössner M, Black RE. Undernutrition as an underlying cause of child deaths associated with diarrhea, pneumonia, malaria, and measles. Am J Clin Nutr. 2004;80:193–198. 15213048 10.1093/ajcn/80.1.193

[18] Ehrhardt S, Burchard GD, Mantel C, Cramer JP, Kaiser S, Kubo M, et al Malaria, Anemia, and Malnutrition in African Children—Defining Intervention Priorities. The Journal of Infectious Diseases. 2006;194:108–114. doi: 10.1086/504688 16741889 10.1086/504688

[19] Kateera F, Ingabire CM, Hakizimana E, Kalinda P, Mens PF, Grobusch MP, et al Malaria, anaemia and under-nutrition: three frequently co—existing conditions among preschool children in rural Rwanda. Malar J. 2015;14. 10.1186/s12936-015-0973-z PMC4635556 26542672

[20] Friedman JF, Kwena AM, Mirekl LB, Kariuki SK, Terlouw DJ, Philips-Howard PA, et al Malaria and nutritional status among pre-school children: Results from cross-sectional surveys in western Kenya. AmJTropMed Hyg. 2005;73 (4):698–704. 16222012

[21] Shikur B, Deressa W, Lindtjørn B. Association between malaria and malnutrition among children aged under-five years in Adami Tulu District, south-central Ethiopia: a case–control study. BMC Public Health. 2016;16:174 doi: 10.1186/s12889-016-2838-y 26895759 10.1186/s12889-016-2838-y PMC4759858

[22] Maketa V, Mavoko HM, Luz RId, Zanga J, Lubiba J, Kalonji A, et al The relationship between Plasmodium infection, anaemia and nutritional status in asymptomatic children aged under five years living in stable transmission zones in Kinshasa, Democratic Republic of Congo. Malaria Journal 2015. 10.1186/s12936-015-0595-5 PMC4336722 25880427

[23] Ahmad SH, Moonis R, Shahab T, Khan HM, Iilani T. Effect of nutritional status on total parasite count in malaria Indian J Pediatr 1985; 52: 285–288. 3910578 10.1007/BF02754860

[24] Mitangala P, D'Alessandro U, Donnen P, Hennart P, Porignon D, Bisimwa Balaluka G, et al Malaria infection and nutritional status: results from a cohort survey of children from 6 to 59 months old in the Kivu province, Democratic Republic of the Congo. Rev Epidemiol Sante Publique. 2013;61:111–120. doi: 10.1016/j.respe.2012.06.404 23489948 10.1016/j.respe.2012.06.404

[25] Deribew A, Alemseged F, Tessema F, Sena L, Birhanu Z, Zeynudin A, et al Malaria and under-nutrition: A community based study among under-five children at risk of malaria, South-West Ethiopia. PLos one. 2010;5 (5). 10.1371/journal.pone.0010775 PMC2874013 20505829

[26] Lehmann D, Howard P, Heywood P. Nutrition and Morbidity: Acute Lower Respiratory Tract Infections, Diarrhoea and Malaria. PNG Med J 2005;48(1–2):87–94. 16894840

[27] Nyakeriga AM, Troye-Blomberg M, Chemtai AK, Marsh K, Williams TN. Malaria and nutritional status in children living on the coast of Kenya. Am J Clin Nutr. 2004;80:1604–1610. 15585775 10.1093/ajcn/80.6.1604

[28] Williams TN, Maitland K, Phelps L, Bennett S, Peto TEA, Viji J, et al Plasmodium Vivax: a cause of malnutrition in young children. QJ Med 1997;90:751–757. 10.1093/qjmed/90.12.751 9536339

[29] Danquah I, Dietz E, Zanger P, Reither K, Ziniel P, Bienzle U, et al Reduced efficacy of intermittent preventive treatment of malaria in malnourished children. Antimicrobial Agents and Chemotherapy. 2009;53 (5):1753–1759. doi: 10.1128/AAC.01723-08 19223620 10.1128/AAC.01723-08 PMC2681506

[30] Prudence MN, D’Alessandro U, Donnen P, Hennart P, Porignon D, Ghislain BB, et al Clinical Malaria and Nutritional Status in Children Admitted in Lwiro Hospital, Democratic Republic of Congo. J Clin Exp Pathol 2012;S3.

[31] Alexandre MAA, Benzecry SG, Siqueira AM, Vitor-Silva S, Melo GC, Monteiro WM, et al The association between nutritional status and malaria in children from a rural community in the Amazonian Region: A longitudinal study. PLoS Negl Trop Dis. 2015;9(4). 10.1371/journal.pntd.0003743 PMC4415998 25928774

[32] CSA. Ethiopian population and housing census. Addis Ababa, Ethiopia: Central Statistical Agency, 2007.

[33] NMA. Adami Tulu and Ziway town annual meteorology data: Ethiopia Meteorology Agency, Hawassa branch. 2016 (Unpublished).

[34] Kloos H, Lindtjørn B. Famine and malnutrtion In: Kloos H, Zein Z, editors. The ecology of health and disease in Ethiopia: Westview press, Boulder and Oxford; 1993.

[35] IFRC. Emergency plan of action Ethiopia: Drought. International Federation of Red Cross and Red Crescent Societes, 2015.

[36] Ersino G, Henry CJ, Zello Gordon A.. Suboptimal feeding practices and high levels of undernutrition among infants and young children in the rural communities of Halaba and Zeway, Ethiopia. Food and Nutr Bull. 2016. 10.1177/0379572116658371 27402640

[37] Deressa W, Loha E, Balkew M, Hailu A, Gari T, Kenea O, et al Combining long-lasting insecticidal nets and indoor residual spraying for malaria prevention in Ethiopia: study protocol for a cluster randomized controlled trial. Trials. 2016;17:20 doi: 10.1186/s13063-016-1154-2 26758744 10.1186/s13063-016-1154-2 PMC4711025

[38] WHO. Guidelines for the treatment of malaria, 3rd edition. Geneva, Switzerland: World Health Organization, 2015.

[39] WHO. Universal access to malaria diagnostic testing: An operational manual. Geneva Switzerland: World Health Organization, 2011.

[40] MOH. National malaria guidelines Addis Ababa, Ethioipa: Ministry of Health; 2012.

[41] WHO. Reliability of anthropometric in the WHO multicentre growth reference study. Acta Pædiatrica. 2006;Suppl 450. 10.1111/j.1651-2227.2006.tb02374.x 16817677

[42] WHO. WHO child growth standards based on length/height, weight and age: WHO multicenter growth references study group. Acta Pædiatrica. 2006. 10.1111/j.1651-2227.2006.tb02378.x 16817681

[43] Erhardt J, Golden M, Seaman J, inventors Emergency nutrition assessment for SMART software 2015.

[44] Vyas S, Kumaranayake L. Constructing socio-economic status indices: how to use principal components analysis. Health Policy Plan. 2006;21:459–468. doi: 10.1093/heapol/czl029 17030551 10.1093/heapol/czl029

[45] Gari T, Loha E, Deressa W, Solomon T, Atsbeha H, Assegid M, et al Anaemia among children in a drought affected community in south-central Ethiopia. PLoS ONE 2017;12(3): e0170898 doi: 10.1371/journal.pone.0170898 28291790 10.1371/journal.pone.0170898 PMC5349654

[46] Twisk J. Applied longitudinal data analysis for epidemiology: A practical guide. United States of America: Cambridge University press; 2003.

[47] MOH. Training course on the managment of acute severe malnutrition: Participant manual. Addis Ababa, Ethiopia: Ministry of Health; 2013.

[48] Alemu A, Abebe G, Tsegaye W, Golassa L. Climatic variables and malaria transmission dynamics in Jimma town, South West Ethiopia. Parasites & Vectors. 2011;4:30. 21366906 10.1186/1756-3305-4-30 PMC3055844

[49] Yisak H, Gobena T, Mesfin F. Prevalence and risk factors for undernutrition among children under five at Haramaya district, Eastern Ethiopia. BMC Pediatrics. 2015;15:212 doi: 10.1186/s12887-015-0535-0 26675579 10.1186/s12887-015-0535-0 PMC4682239

[50] McGregor IA. Malaria: Nutritional Implications. Review of Infectious Diseases. 1982;4 (4). 10.1093/4.4.798 6812196

[51] Calder PC, Jackson AA. Undernutrition, infection and immune function. Nutrition Research Reviews. 2000;13:3–29. doi: 10.1079/095442200108728981 19087431 10.1079/095442200108728981

[52] Padonou G, Port AL, Cottrell G, Guerra J, Choudat I, Rachas A, et al Factors associated with growth patterns from birth to 18 months in a Beninese cohort of children. Acta Tropica. 2014;135:1–9. doi: 10.1016/j.actatropica.2014.03.005 24674879 10.1016/j.actatropica.2014.03.005

[53] UNICEF. Improving child nutrition: The achievable imperative for global progress. Unicef: 2013.

[54] Schaible UE, Kaufmann SHE. Malnutrition and infection: Complex mechanisms and global impacts. PLoS Med. 2007;4(5):e115 doi: 10.1371/journal.pmed.0040115 17472433 10.1371/journal.pmed.0040115 PMC1858706

[55] Ferreira EdA, Alexandre MA, Salinas JL, Siqueira AMd, Benzecry SG, Lacerda MVGd, et al Association between anthropometry-based nutritional status and malaria: a systematic review of observational studies. Malar J 2015;14:346 doi: 10.1186/s12936-015-0870-5 26377094 10.1186/s12936-015-0870-5 PMC4574180

[56] Golassa L, Baliraine FN, Enweji N, Erko B, Swedberg G, Aseffa A. Microscopic and molecular evidence of the presence of asymptomatic Plasmodium falciparum and Plasmodium vivax infections in an area with low, seasonal and unstable malaria transmission in Ethiopia. BMC Infectious Diseases. 2015;15:310 doi: 10.1186/s12879-015-1070-1 26242405 10.1186/s12879-015-1070-1 PMC4526179

[57] Tadesse F, Hoogen vd, Lanke K, Schildkraut J, Tetteh K, Aseffa A, et al The shape of the iceberg: quantification of submicroscopic Plasmodium falciparum and Plasmodium vivax parasitaemia and gametocytaemia in five low endemic settings in Ethiopia. Malar J. 2017;16(1):99 doi: 10.1186/s12936-017-1749-4 28253867 10.1186/s12936-017-1749-4 PMC5335517

[58] Golassa Lemu, Email author, Enweji Nizar, Erko Berhanu, Aseffa Abraham, Swedberg Göte. Detection of a substantial number of submicroscopic Plasmodium falciparum infections by polymerase chain reaction: a potential threat to malaria control and diagnosis in Ethiopia. Malar J. 2013;12:352 doi: 10.1186/1475-2875-12-352 24090230 10.1186/1475-2875-12-352 PMC3850638

[59] CSA. Ethiopian Demographic and Health Survey. Addis Ababa: Central Statistical Agency, 2016.

[60] Egata G, Berhane Y, Worku A. Seasonal variation in the prevalence of acute undernutrition among children under five years of age in east rural Ethiopia: a longitudinal study. BMC Public Health. 2013;13:864 doi: 10.1186/1471-2458-13-864 24047474 10.1186/1471-2458-13-864 PMC3851835

[61] Degarege A, Legesse M, Medhin G, Animut A, Erko B. Malaria and related outcomes in patients with intestinal helminths: a cross-sectional study. BMC Infectious Diseases 2012;12:291 doi: 10.1186/1471-2334-12-291 23136960 10.1186/1471-2334-12-291 PMC3519704

[62] Hailu T. Current prevalence of intestinal parasites emphasis on hookworm and schistosoma mansoni infections among patients at Workemeda Health Center, Northwest Ethiopia. Clin Microbial 2014;3: 4.

