# Supplementary figures and images for: Malaria increased the risk of stunting and wasting among young children in Ethiopia: Results of a cohort study
Source: PLoS One. 2018 Jan 11;13(1):e0190983. doi: 10.1371/journal.pone.0190983 (PMC5764317; doi:10.1371/journal.pone.0190983)

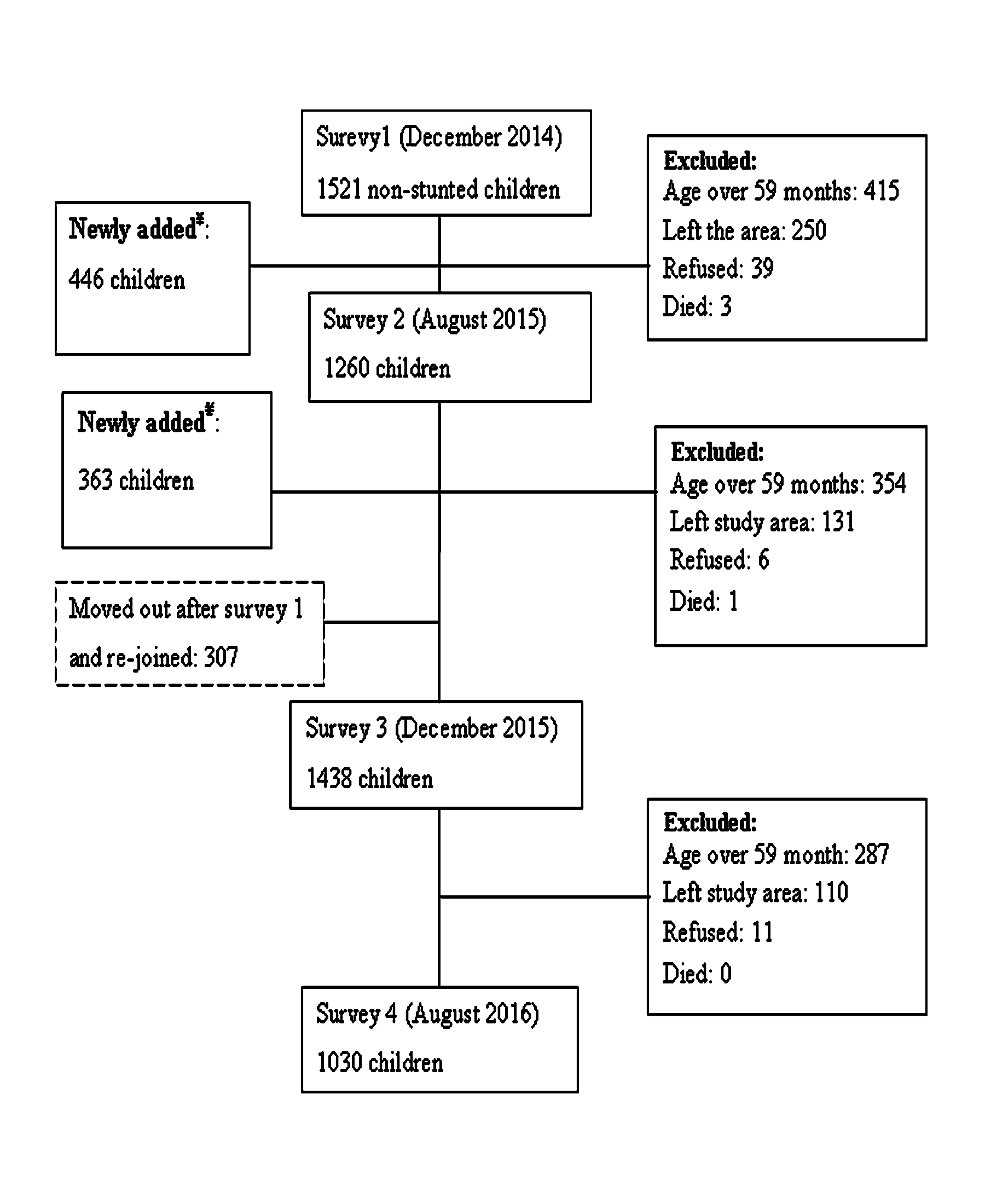

Supplement: S1 Fig — ¥: Newly added include newborn children aged 6 month and above during the survey and newcomers. (TIF) [file pone.0190983.s001.TIF]

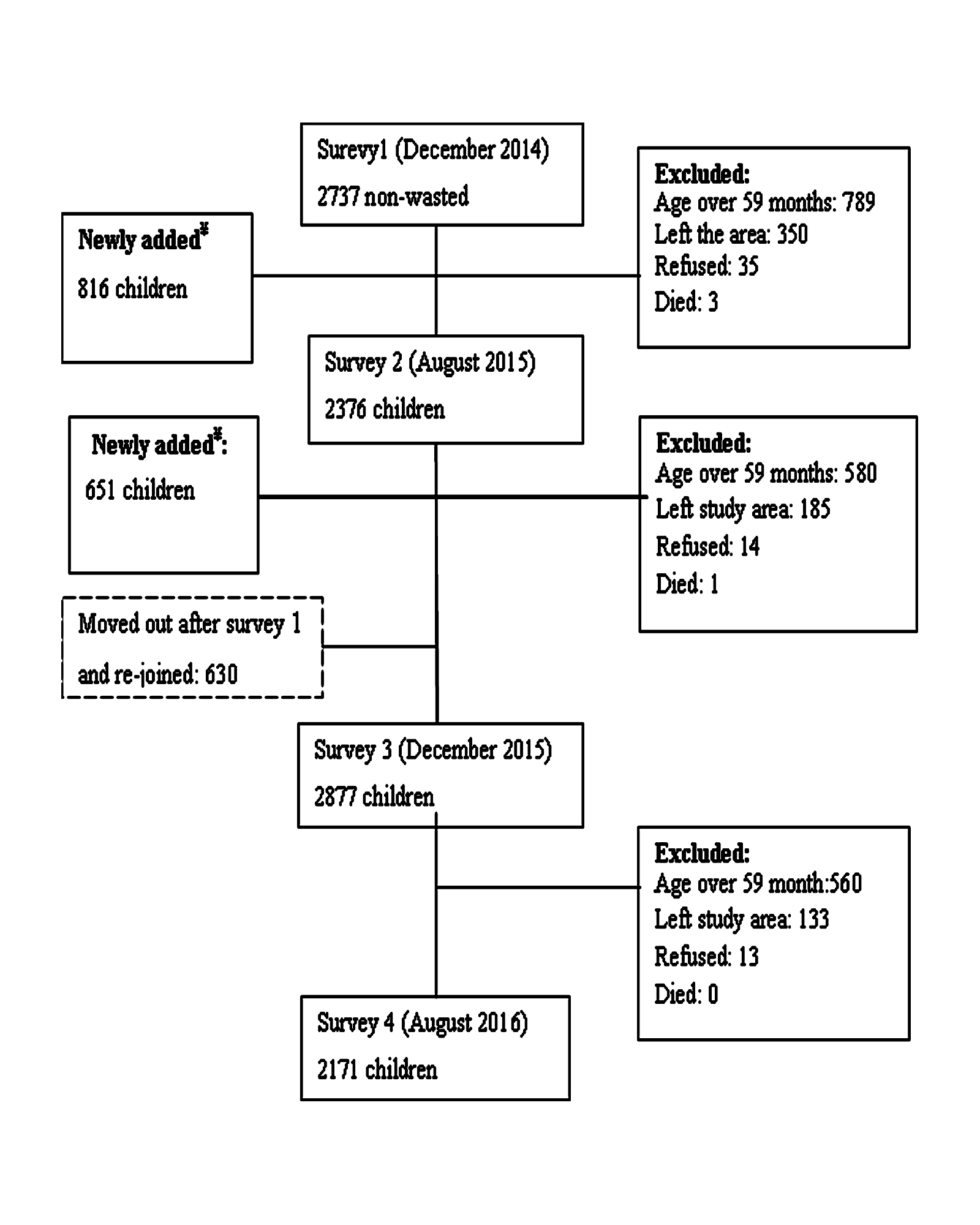

Supplement: S2 Fig — ¥: Newly added include newborn children aged 6 month and above during the survey and newcomers. (TIF) [file pone.0190983.s002.TIF]
